# Solution Blow Spinning of Polycaprolactone—Rheological Determination of Spinnability and the Effect of Processing Conditions on Fiber Diameter and Alignment

**DOI:** 10.3390/ma14061463

**Published:** 2021-03-17

**Authors:** Katarzyna Czarnecka, Michał Wojasiński, Tomasz Ciach, Pawel Sajkiewicz

**Affiliations:** 1 Institute of Fundamental Technological Research, Polish Academy of Sciences, Pawinskiego 5b, 02-106 Warsaw, Poland; psajk@ippt.pan.pl; 2 Faculty of Chemical and Process Engineering, Warsaw University of Technology, Warynskiego 1, 00-645 Warsaw, Poland; michal.wojasinski@pw.edu.pl (M.W.); tomasz.ciach@pw.edu.pl (T.C.); 3 Warsaw University of Technology, CEZAMAT, Poleczki 19, 02-822 Warsaw, Poland

**Keywords:** solution blow spinning, rheology, image analysis, nanofibers, fiber alignment, biodegradable nanofibers

## Abstract

The growing popularity of solution blow spinning as a method for the production of fibrous tissue engineering scaffolds and the vast range of polymer–solvent systems available for the method raises the need to study the effect of processing conditions on fiber morphology and develop a method for its qualitative assessment. Rheological approaches to determine polymer solution spinnability and image analysis approaches to describe fiber diameter and alignment have been previously proposed, although in a separate manner and mostly for the widely known, well-researched electrospinning method. In this study, a series of methods is presented to determine the processing conditions for the development of submicron fibrous scaffolds. Rheological methods are completed with extensive image analysis to determine the spinnability window for a polymer–solvent system and qualitatively establish the influence of polymer solution concentration and collector rotational speed on fiber morphology, diameter, and alignment. Process parameter selection for a tissue engineering scaffold target application is discussed, considering the varying structural properties of the native extracellular matrix of the tissue of interest.

## 1. Introduction

In recent decades, submicron fibers have been steadily gaining the interest of researchers and the industry alike [1]. The unique properties and tailorable microstructure of submicron fibers make them useful in a range of applications, such as wound dressings [2], drug delivery platforms [3,4], energy materials [5,6], sensors [7], and filtration materials [8,9,10]. Submicron fibers of biocompatible and biodegradable polymers are also of particular interest in the field of tissue engineering and regenerative medicine [11,12]. Due to their structural similarity to the natural extracellular matrix (ECM), fibrous mats of such polymers can serve as synthetic scaffolds, mimicking the native ECM [13]. Tissue engineering scaffolds aim to provide cells with mechanical, chemical, topographical, and other physical cues to facilitate cell securing, differentiation, and growth, allowing for extensive functional tissue regeneration [14,15].

However, the scaffolds need to be carefully designed to exhibit the desired properties and provide an adequate environment to facilitate cell growth. Therefore, the use and development of a method enabling both the rapid and extensive selection of process parameters directly influencing fibrous scaffold morphology are in order for the structural mimicry approach [15].

Biodegradable polymer mats made from randomly aligned fibers structurally similar to the native ECM of, e.g., skin or bone tissue [16,17,18,19] or highly aligned fibers characteristic for the anisotropic structure of, e.g., nervous or myocardial muscle tissue [20,21,22,23,24] can be successfully produced using the solution blow spinning (SBS) method with controlled process parameters, as shown in this study. Solution blow spinning is a relatively novel method for the production of submicron polymer fibers, currently gaining significant interest as a rapid, safe, and attractive alternative to the decades-old, widely used electrospinning [25,26,27].

In SBS, concentric nozzles are used, with the polymer solution being extruded through the inner nozzle and high-pressure gas released through the outer nozzle. The compressed gas with a high enough pressure causes a polymer solution jet discharge from the tip of a Taylor cone at the exit of the inner nozzle. The jet is then accelerated towards the target over the working distance. As the solvent evaporates, polymer fibers are left and can be collected on essentially any target. In contrast, electrospinning relies on a high voltage difference between the target and the polymer outlet [28,29]. Even though the SBS method is promising in its potential for significantly more rapid fiber production rates [30,31] and a wider range of available polymer–solvent systems, comprehensive research still needs to be conducted to establish the relationships between processing parameters and the fibrous material structure [27,32].

SBS is used to produce fibrous polymer mats with a wide range of morphologies, controlled by a number of process parameters [32,33,34]. The characteristics of solution blow spun polymer fibrous mats, such as average fiber diameter and diameter distribution, fiber morphology, alignment, and porosity, are affected by the process parameters, such as polymer solution concentration, solvent type, and polymer–solvent molecular interactions, solution viscosity, air pressure, polymer solution feed rate, and collector rotational speed [35,36,37].

It has been established that the formation of uniform fibers is dependent on the polymer solution concentration, which influences solution viscosity. Polymer macromolecule extension and entanglement vary between dilute, semi-dilute unentangled, and semi-dilute entangled polymer concentration regimes [35,38]. In principle, low to no interaction between polymer chains leads to spraying and solidification of the solution droplets as polymer film on the surface of the collector (dilute regime), low interaction and entanglement between macromolecules cause so-called beads to appear in the fiber network (semi-dilute unentangled regime) [39], and high macromolecular entanglement in the polymer solution causes the fiber formation process to stabilize and yield uniform fibers [40,41]. Critical values limiting these regimes constitute a so-called spinnability window, or a set of conditions where uniform fiber spinning should be possible. Therefore, rheological measurements may be used to assess the solution concentration range of a given polymer-solvent system.

Rheological measurements can be used to gain insight into the microstructure of rheologically complex fluid systems, which can be easily influenced by the application of relatively low stresses [42]. Most of these systems contain irregularly shaped particles (such as entangled polymer chains), which, at first, resist the applied stress, resulting in high apparent viscosities at low shear rates, approaching theoretical zero shear rate viscosity. The application of more stress causes the particles to deform or, in the case of coiled or entangled polymer macromolecules, straighten out, uncoil, or unentangle. Such microstructural changes correspond to bulk flow in the system, resulting in lower apparent viscosity as shear increases [43]. This phenomenon allows for the formation and acceleration of a polymer solution jet, which solidifies into fibers as it crosses the working distance between the tip of the needle through which the solution is supplied and the collector surface [44]. In the case of SBS, the source of the shear forces is the working gas pressure [32,33].

This study aims to establish an easy-to-use, comprehensive protocol for the selection of solution blow spinning process parameters for the fabrication of non-woven fibrous mats with varying desired average fiber diameters and degrees of alignment, as well as uniform fiber morphology. Such information might prove valuable in the design of synthetic, acellular tissue engineering scaffolds for a wide range of target tissues with varying native ECM morphologies. Poly-ε-caprolactone (PCL) was chosen as a biodegradable, biocompatible polymer with good viscoelastic properties, widely used for tissue engineering applications [45,46].

## 2. Materials and Methods

### 2.1. Materials

Polymer solutions for the fabrication of fibrous samples and rheological measurements were prepared using poly-ε-caprolactone (PCL, Merck KGaA, Darmstadt, Germany), with a number average of M_n_ = 80,000 Da and M_n_ = 45,000 Da for comparative rheological measurements. 2,2,2-trifluoroethanol (TFE, Tokyo Chemical Industry, Tokyo, Japan) was used as the solvent. TFE was chosen as a solvent based on previous research experience, where fibers produced using TFE were found to be the most uniform in shape and size, showed the least bundling, and had smooth surfaces at the magnifications used in scanning electron microscopy.

### 2.2. Preparation of Polymer Solutions

Polymer solutions were prepared using an analytical scale with the weight/weight (*w/w*) concentrations in the range of 0.5–9%, with an increment of 1% in the 1–9% range. All further polymer solution concentration values are expressed as % *w/w*. The PCL pellets and solvent were placed in a clean plastic container, sealed with parafilm, and left for 24 h at 25 °C on a magnetic stirrer for the polymer to completely dissolve, leaving a clear, homogenous solution.

### 2.3. Viscometry

Rheological measurements of the polymer solutions were carried out for the full range of concentrations described above, using a cone-plate Brookfield (HADV-III Ultra) viscometer (Brookfield, Middleboro, MA, USA), cones CP-40 and CP-52. As the polymer solution was expected to exhibit non-Newtonian shear-thinning properties, the measurements were performed in sets of time steps, with a range of shear rates from 200 to 1 s^−1^ and corresponding shear stress values measured for each solution.

Viscosity measurements served to establish the correlation between polymer solution viscosity and spun fiber morphology. Information on rheological properties of the polymer solution can be used to predict fiber morphology and establish the ”spinnability window”, i.e., a set of spinning process conditions where submicron fiber formation is possible. The minimum polymer solution concentration value to allow for macromolecular entanglement in a given polymer–solvent system is known as the critical overlap concentration C*, a boundary value between dilute and semi-dilute polymer solution concentration regimes [47]. In dilute polymer solutions or solutions with poor solvents, polymer chains do not generally overlap [41].

As the solutions of PCL in TFE are shear-thinning liquids, zero shear rate viscosity, $\eta_{0}$, was used as a property characterizing liquid viscosity, commonly used to indicate polymer solution spinnability [29,48,49]. As it is often impossible in practice to measure the viscosity of liquids at a very low shear rate, zero shear viscosity was calculated by extrapolating apparent viscosities measured for a range of higher shear rates, within the measurement capabilities of the rheometer, to shear rate approaching zero [47]. The Carreau regression model [50] was used to extrapolate experimental data to zero shear rate viscosity, as it is found to be in good accordance with experimental data for linear polymer melts and solutions [51,52].

PCL with an average M_n_ = 45,000 Da was also used to prepare solutions for comparative rheological measurements with the same solution preparation protocol and polymer concentration values as above. The purpose was to characterize the average macromolecular conformation of the polymer in the solution by calculating the appropriate Mark–Houwink Equation (1) parameter a. The Mark–Houwink equation correlates the intrinsic viscosity of a polymer solution to the molecular weight of the polymer [53,54].

(1) $$\left[\eta \right]=KM^{a}$$

where [η] is the intrinsic viscosity of the polymer solution, K and a are Mark–Houwink constants, depending on the type of polymer–solvent system and measurement temperature, and M is the molecular weight of the polymer.

The parameter *a* has a defined physical meaning—it is a function of polymer macromolecule geometry. Its values vary from 0.5 to 2, with 0.5 corresponding to a rigid sphere, 0.5–0.8—a random coil, and 0.8–2—a rigid or rod-like chain. Macromolecules can appear in all these shapes; however, the parameter *a* indicates an average configuration, depending on the solvent. Lower values are typical for poor solvents, where the polymer macromolecule segments strongly attract each other, causing them to curl. Conversely, a good solvent will be able to overcome the intramolecular attraction, penetrate the curled macromolecule, and cause the chains to extend significantly [55], allowing them to overlap and entangle—provided the polymer concentration is high enough—which is crucial for the fiber formation processes, such as electrospinning and solution blow spinning [56]. Therefore, the parameter *a* can be considered as an indicator of solvent goodness in a given application [57].

Intrinsic viscosity is a measure of the influence of the solute on solution viscosity. It is defined as the limit of reduced viscosity $\eta_{red}$ at zero polymer concentration *c* and calculated from multi-concentration measurements using the Huggins Equation (2) and its linear approximation Equation (3):

(2) $$\eta_{red}=\frac{\eta -\eta_{solvent}}{c\eta_{solvent}}=\left[\eta \right]+k_{H}{\left[\eta \right]}^{2}c$$

(3) $$\left[\eta \right]=\underset{c\to 0}{\lim}\eta_{red}=\underset{c\to 0}{\lim}\frac{\eta -\eta_{solvent}}{c\eta_{solvent}}$$

where η is the apparent viscosity of a solution at a given shear rate, $\eta_{solvent}$ is the viscosity of the solvent, and $k_{H}$ is the Huggins constant [58,59].

### 2.4. Solution Blow Spinning

The solution blow spinning process was carried out using an in-house system, constructed similarly to [34,60], with the syringe pump, concentric nozzle system, and collector placed in a fume hood for better control of ambient parameters and personal protection purposes. A basic scheme of the in-house system used for fibrous sample preparation can be found in [25].

The polymer solution was supplied with a syringe pump at a constant flow rate of 30 mL/h, connected to a system of two concentric nozzles. Compressed air was used as the working gas and supplied through the outer nozzle with an inlet pressure of 0.1 MPa using an air pump. A cylindrical rotational stainless-steel collector with a diameter of 12.5 mm was used, with the rotational speeds in the range of 3000 to 9000 rpm, with an increment of 1000 rpm. Additionally, samples were collected from the surface of a static collector (denoted as 0 rpm) and a static stainless-steel plate (30 cm × 30 cm), placed perpendicular to the solution outlet needle. The distance from the nozzle to the collector surface and the plate face was 30 cm. Nozzle diameters were as follows: inside diameter of the outer nozzle—4.0 mm; outside diameter of the inner nozzle—1.0 mm; inside diameter of the inner nozzle—0.8 mm. Air humidity inside the fume hood was 23% ± 2% and the temperature was 25 ± 3 °C. The collector surface was wrapped tightly with aluminum foil to facilitate sample removal, cutting, and transportation. Table 1 presents the collector rotational speed converted to the linear speed of a point on the surface of the collector, which can be interpreted as the fiber uptake speed.

### 2.5. Sample Imaging

Samples for scanning electron microscopy (SEM) imaging were cut out in 3 mm × 3 mm squares from randomly chosen locations on the mats with a sharp blade, then sputter-coated with a 15 nm gold layer. The thickness of the gold layer was included in the measured fiber diameter values. A scanning electron microscope (Phenom Pure, Phenom-World, Eindhoven, Netherlands) was used to acquire images at two magnifications, with at least ten randomly selected images for each magnification per sample chosen for digital image analysis.

### 2.6. Image Analysis

Quantitative image analysis was performed using the Fiji distribution of the ImageJ software [61]. Fiber diameters were measured manually. In total, 150 diameters were measured for each sample, i.e., each set of polymer solution concentration and collector rotational speed. This amounted to 1200 fibers measured for samples produced from each polymer solution concentration.

Fiber alignment measurements in relation to processing parameters (solution concentration and collector rotational speed) served to establish a protocol for the fast production of scaffolds for the tissue of interest with known native ECM morphology. Fiber alignment was calculated as per the following protocol: fiber orientation was calculated using the Fiji Directionality plugin to ImageJ, providing results in the form of a share of total fibers within a 1° angle from 0° to 180°. The Directionality plugin uses Fourier components analysis [62], in which an image is divided into squares, and a periodic pattern of the orientation of structures in the image is created using Fourier transform. The resulting Fourier power spectra are then analyzed in polar coordinates and power is measured for each angle [63,64].

The raw data were fitted to Pearson VII distributions in Origin 2019 [65], and the calculated full width at half maximum (FWHM) values were averaged over ten images from each sample produced with a given set of polymer solution concentration and collector rotational speed parameters. The averaged value represents the inverse of fiber alignment—low FWHM values mean narrow fiber direction distributions, representing highly aligned fiber structures. Conversely, large FWHM values mean low fiber alignment, with fibers present in a wide range of directions.

## 3. Results and Discussion

### 3.1. Viscometry

#### 3.1.1. Spinnability Window

**Figure 1:**
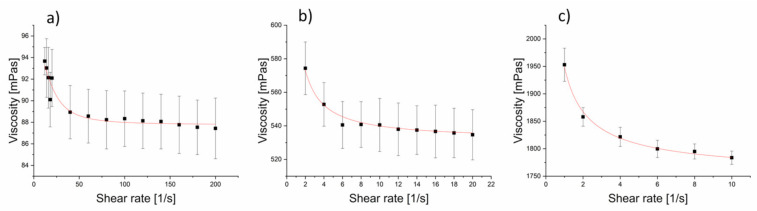
Carreau model fits of apparent viscosity vs. shear rate for 80 kDa PCL solutions in (**a**) 3%, (**b**) 6%, and (**c**) 9% concentrations.

Apparent solution viscosities were measured in a wide range of shear rates, with five measurement series for each solution sample. Data fitting of average apparent viscosity values was performed using the Origin software. The Carreau model was successfully used to determine zero shear viscosities of all the prepared PCL 80 kDa solutions, with R^2^ > 0.92 averaging over all the flow curve fits. Figure 1 shows plots of average apparent viscosity vs. shear rate fitted to the Carreau model for polymer solution concentrations of 3%, 6%, and 9% PCL in TFE.

**Figure 2:**
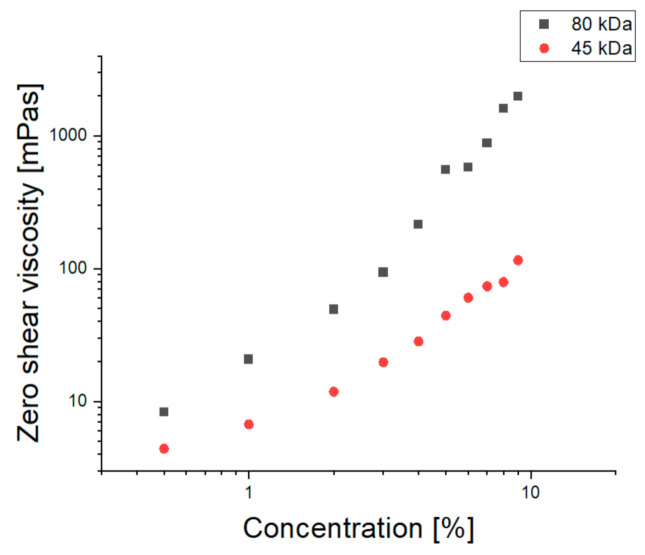
Changes in zero shear viscosity determined using the Carreau model for TFE solutions of PCL with a molecular weight of 80,000 and 45,000 Da. Error bars are covered by the symbols.

It was observed that zero shear viscosity of the solutions was dependent on the solution concentration for PCL with both number average molecular weights in different solution concentration regimes (Figure 2). The changes in zero shear viscosity in relation to the solution concentration were similar for both polymer molecular weights, with the differences between particular solution concentration regimes being more pronounced for the 80 kDa PCL, which is consistent with the theoretical explanation of the phenomenon being based on the macromolecular structure of the solution.

Different power-law regimes for zero shear viscosity vs. polymer concentration were calculated from rheological measurements and cross-examined with SEM images showing different fiber morphologies, depending on the solution concentration. Solution concentration regimes —critical overlap concentration (C*) and critical entanglement concentration (C_e_)—were calculated for the 80 kDa PCL, used to produce fibrous samples. These values were calculated as the polymer solution concentration values at the intersections of linear fits of zero shear viscosity data in the dilute/semi-dilute unentangled and semi-dilute unentangled/semi-dilute entangled regimes, for C* and C_e,_ respectively.

**Figure 3:**
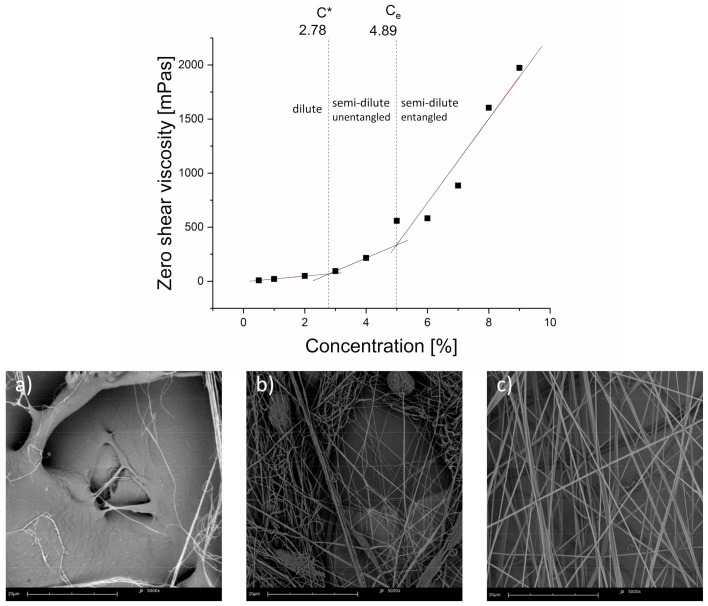
Zero shear rate viscosity of PCL (M_n_= 80,000 D(a) vs. PCL solution concentration. Dilute, semi-dilute, unentangled, and semi-dilute entangled regimes are shown, along with critical overlap concentration (C*) and critical entanglement concentration (C_e_) values. Representative SEM micrographs of the samples, prepared using solutions from the corresponding concentration regimes are shown below: (**a**) dilute regime, 2% solution, polymer film with occasional fibers; (**b**) semi-dilute unentangled regime, 4% solution-uniform and non-uniform fibers with beads; (**c**) semi-dilute entangled regime, 8% solution-uniform fibers. Error bars are covered by the symbols.

For dilute and semi-dilute unentangled concentration regimes, the average R^2^ was 0.96, indicating a good linear fit of the data. Concentration regimes and their limit values, as well as representative SEM micrographs of samples produced from corresponding polymer solutions in the concentration regimes, are shown below (Figure 3). Images (a), (b), and (c) in Figure 3 show the presence of sprayed film, beaded and non-uniform fibers, and uniform fibers, respectively.

#### 3.1.2. Macromolecular Conformation

**Figure 4:**
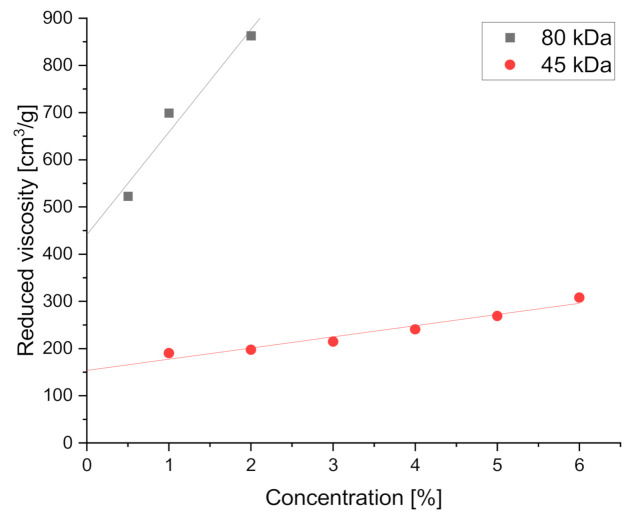
Reduced viscosity vs. PCL/TFE solution concentration with linear fits of reduced viscosity at a shear rate of 30 (1/s).

The molecular conformation of polymers in solutions is affected by the polymer–solvent interactions, which, in turn, can be related to Mark–Houwink equation parameter *a*. The intrinsic viscosity of PCL solutions with two molecular weights, 45 and 80 kDa, was calculated from reduced viscosities using the Huggins method, as the intercept of the linear approximation of the Huggins equation. Reduced viscosity values, as shown in Figure 4, were calculated from the measured apparent viscosities at a shear rate of 30 s^−1^. Intrinsic viscosity was 153.8 ± 10.4 and 441.2 ± 61.8 cm^3^/g for 45 and 80 kDa PCL solutions, respectively. Note that for the 80 kDa PCL, only data from the dilute polymer concentration regime were taken into account to avoid the significant influence of macromolecular entanglement on the calculated parameter *a*, which applies to an average shape of an unentangled molecule, calculated using the intrinsic viscosity value. TFE viscosity value of 1.78 mPa∙s was taken from the literature [66].

Table 2 shows the results of the Mark–Houwink equation parameter calculations. The parameter *a* value was 1.83 ± 0.16, which indicates an average rigid or rod-like shape of polymer macromolecules, significantly extended in the polymer solution with a good solvent. The result is consistent with the literature data, indicating that using TFE as a solvent for PCL fiber spinning led to the formation of uniform fibers [67,68].

### 3.2. Digital Image Analysis

#### 3.2.1. Qualitative Image Analysis

SEM imaging served for qualitative and quantitative assessment of the samples. The resulting images showed a varied morphology of the fibrous mats, changing with the processing parameters. Beaded fibers, i.e., fibers with a so-called “beads-on-a-string” morphology—varying in cross-sectional shape and area along the fiber length—were not found for samples produced from solutions with concentrations above 4%. This indicates a smooth, steady, and constant fiber formation process for solutions with polymer concentrations above the critical entanglement concentration, C_e,_ as seen in Figure 3. The C_e_ = 4.89% value was empirically derived from data shown in Figure 2 and Figure 3, as the polymer solution concentration value at the intersection of the linear fits of the data in the semi-dilute unentangled and semi-dilute entangled polymer solution concentration regimes. Similarly, samples produced from polymer solutions below the 5% polymer concentration exhibited a morphology with uniform and non-uniform fibers with a varying share of beaded fibers and few spray film patches. This observation is consistent with the rheological measurements, which place the critical overlap concentration, C*, at 2.78%.

Therefore, C_e_ = 4.89% can be considered the lower spinnability window concentration value for the PCL/TFE solvent system in given experimental conditions. For this setup, 9% PCL in TFE is considered the upper spinnability window concentration value, as the jet formation process becomes unstable with higher polymer solution concentrations, causing the solvent to rapidly evaporate at the needle tip. This prevents the Taylor cone and jet formation and results in non-uniform fibers with large droplets of the semi-solidified solution being pushed out of the needle at irregular intervals.

#### 3.2.2. Fiber Diameter Measurements

**Figure 5:**
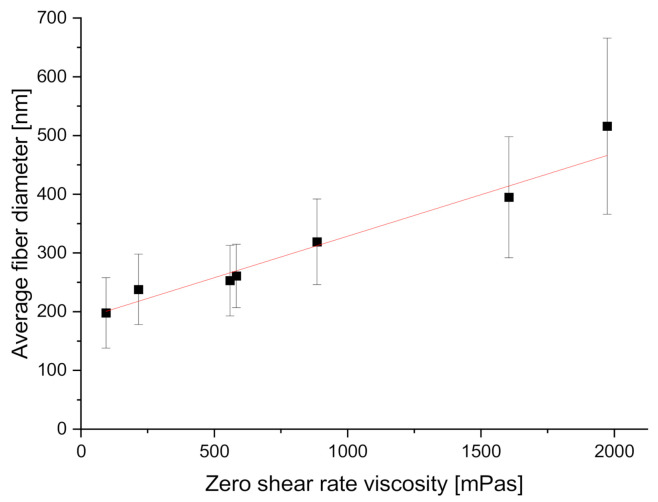
Changes in average fiber diameter, measured across all samples produced from solutions with each concentration, depending on solution zero shear rate viscosity.

**Figure 6:**
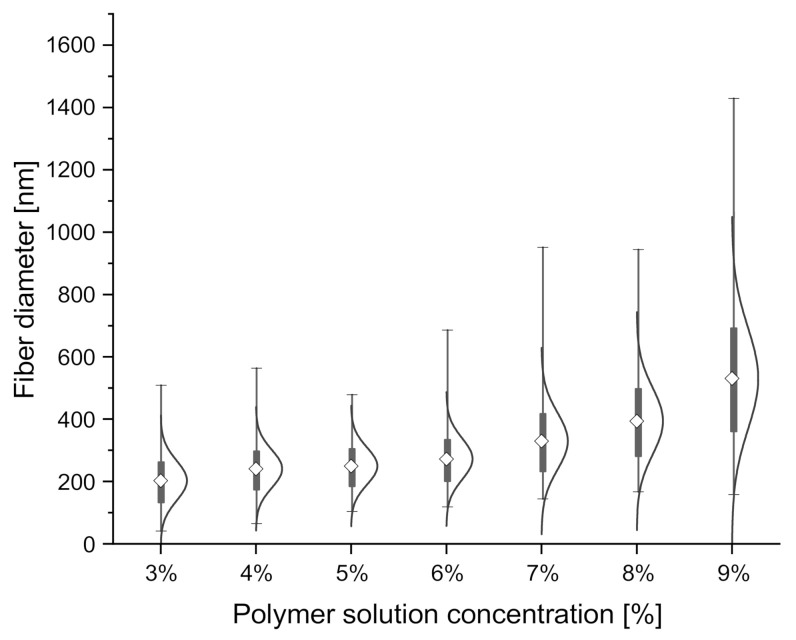
Fiber diameter and polymer solution concentration with average ± SD box values and normal diameter distributions. Min–max values are shown as horizontal strikes.

**Figure 7:**
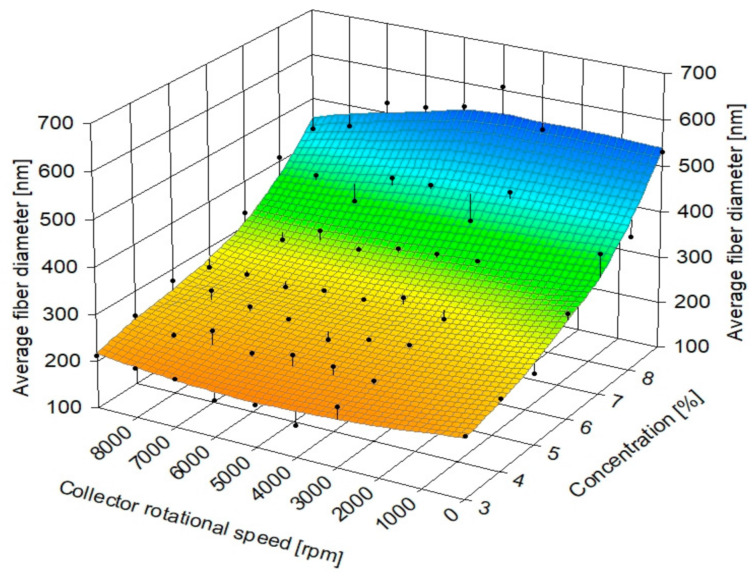
Changes in average fiber diameter with collector rotational speed for every polymer solution concentration used to produce fibrous samples.

**Figure 8:**
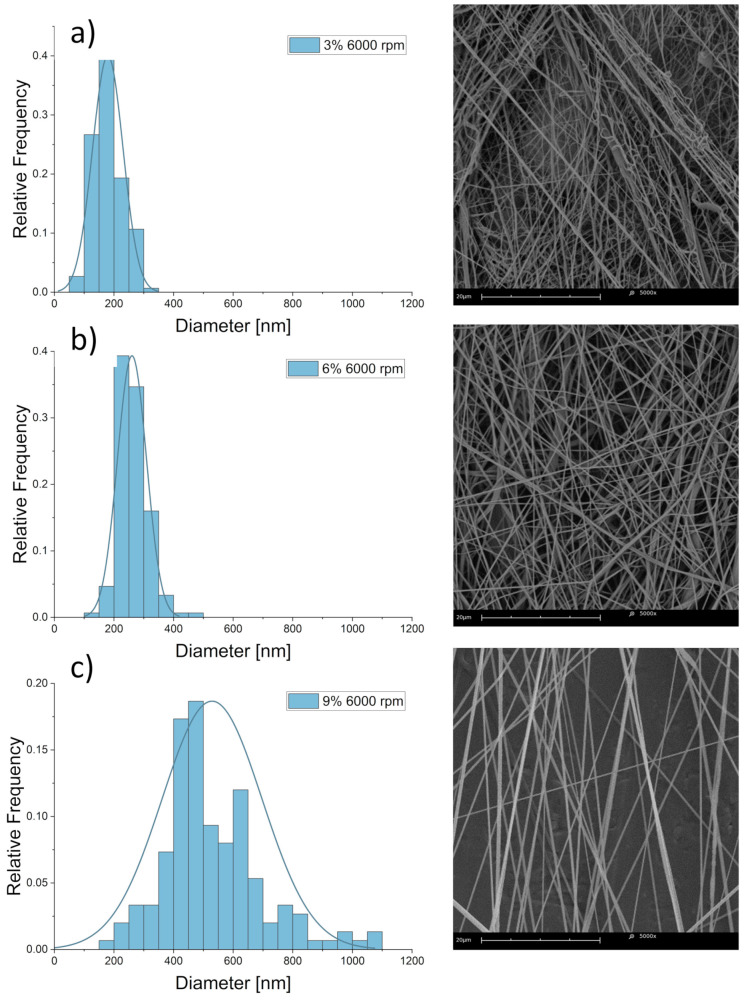
Relative frequency histograms of fiber diameter distributions and respective representative SEM micrographs for samples spun from (**a**) 3%, (**b**) 6%, and (**c**) 9% PCL solutions. Collector rotational speed—6000 rpm.

The chosen parameters allowed for the production of fibers with average ± SD diameters from 198 ± 60 nm at 3% polymer solution concentration to 516 ± 150 nm at 9%. The results of fiber diameter and zero shear viscosity measurements are shown below (Table 3). The coefficient of variation was used to show the extent of variability in fiber diameter distributions. Fiber diameter distribution varied non-linearly, with the most considerable differences visible in the diameters of fibers produced from polymer solutions with the lowest and the highest concentrations. Average fiber diameter was found to non-linearly increase with polymer solution concentration, as shown in a violin plot with kernel-smoothed distributions, with average ± SD (Figure 5), which is consistent with the literature [69,70]. Fiber diameter was also found to linearly depend on zero shear rate viscosity [40] (Figure 6), with R^2^ = 0.95 for the linear fit of the data. No significant influence of collector rotational speed on average fiber diameter was found, as shown in Figure 7, which is also consistent with the literature [30]. Locally estimated scatterplot smoothing (LOESS) [71], a non-parametric regression method, was used in data surface fitting (R^2^ = 0.9). Examples of fiber diameter relative frequency histograms with corresponding representative SEM images are shown in Figure 8. The diameter measurements place the fibers almost exclusively within the native structural ECM proteins diameter range of 50–500 nm. This size range is considered to be the most adequate for tissue engineering, as such fibers closely mimic the cellular environment [72].

#### 3.2.3. Fiber Alignment Assessment

**Figure 9:**
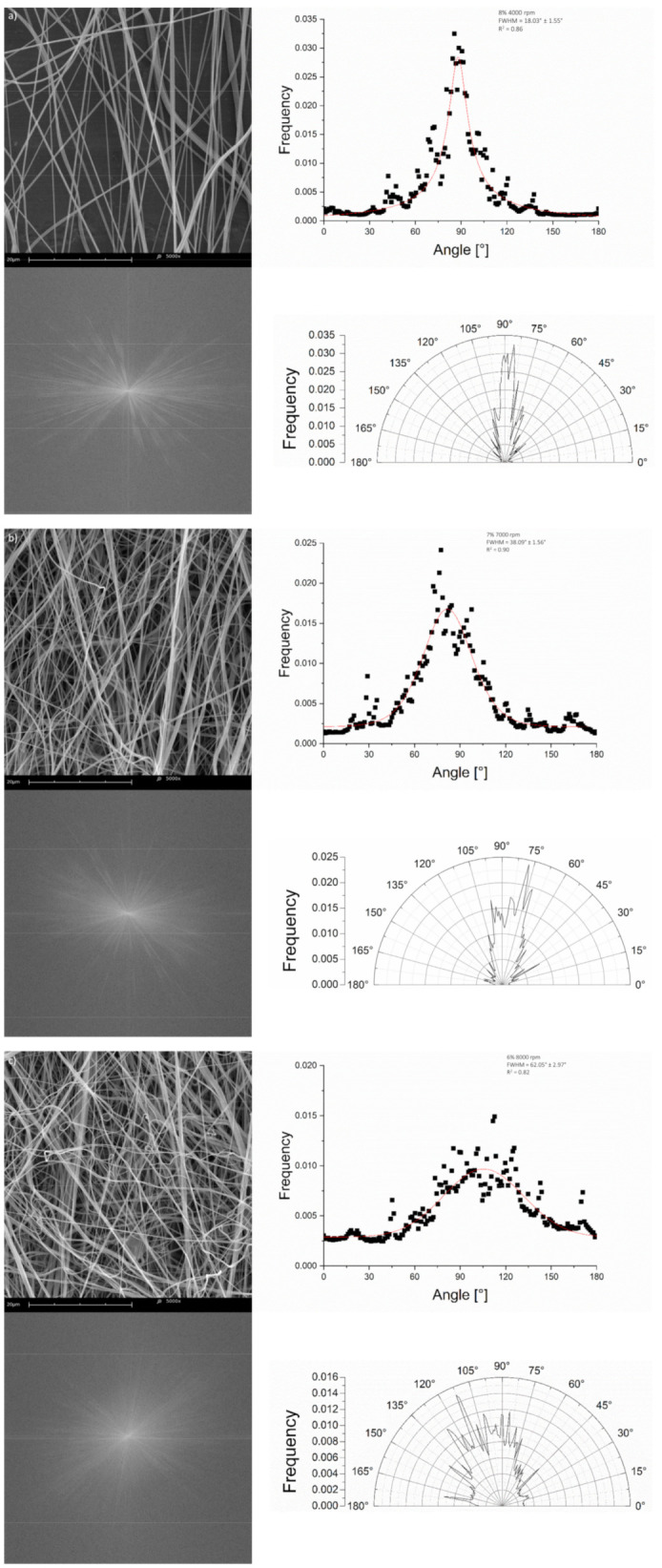
Examples of fiber angle distributions fitted to the Pearson VII function with representative SEM images, FFT power spectra, and polar coordinate directionality diagrams for (**a**) highly aligned fibers, (**b**) moderately aligned fibers, and (**c**) poorly aligned fibers. FWHM and R^2^ fit values are included.

Figure 9 shows a comparison of Pearson VII function fits between representative highly aligned (Figure 9a), moderately aligned (Figure 9b), and poorly aligned (Figure 9c) fiber samples, along with examples of corresponding SEM images, Fast Fourier Transform (FFT) power spectra, and polar coordinate diagrams.

**Figure 10:**
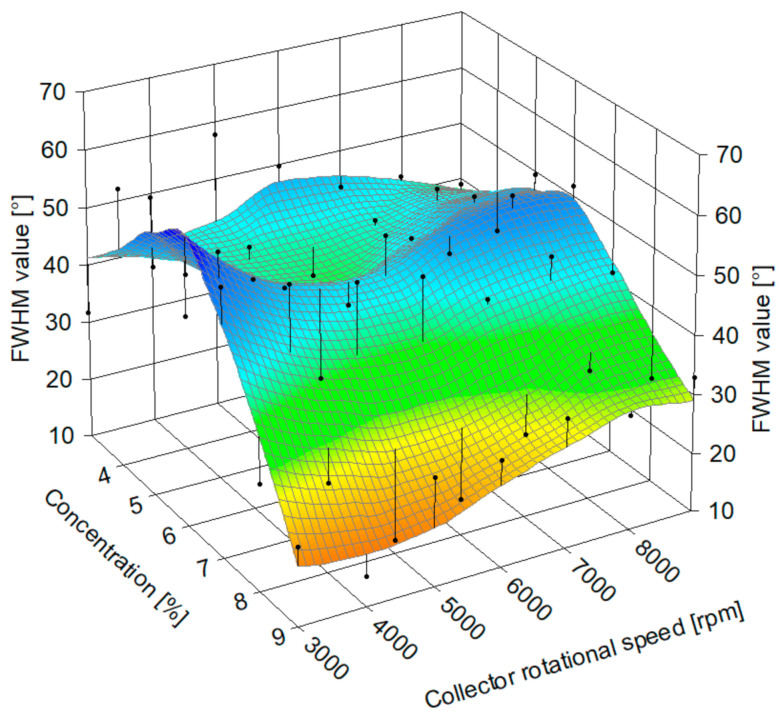
The relation between collector rotational speed, polymer solution concentration, and fiber alignment expressed as FWHM values of Pearson VII fits to fiber directionality histograms.

A non-linear relationship was found between the polymer solution concentration, collector rotational speed, and fiber alignment, expressed as the full width half maximum (FWHM) value of the peak in the fitting of experimental fiber directionality histogram to the Pearson VII function (Figure 10). Higher FWHM values correspond to a wider fit to the orientation data and, therefore, to less aligned fibers. The surface diagram excludes samples gathered on a static collector, as they are by necessity gathered on a base with a different geometry. LOESS was used in data surface fitting. The lowest FWHM value was 12.22° ± 1.01° for 8% solution concentration at 5000 rpm collector rotational speed, and the highest was 60.55° ± 4.48° for 5% at 3000 rpm.

No significant influence of collector rotational speed on the fiber alignment was found for solutions within the semi-dilute unentangled regime—3% and 4% polymer concentration. This is consistent with the results of rheological measurements and qualitative SEM image assessment, as a number of fibers in these samples are visibly finer than the others, as well as interconnected between beads. Therefore, it can be assumed that their degree of alignment is more significantly influenced by local fiber–fiber and fiber–bead connections than the changes in fiber uptake speed (collector rotational speed), as is the case in samples spun from solutions within the semi-dilute entangled regime, with larger, heavier fibers.

For samples with uniform fibers, produced from polymer solutions with concentrations higher than critical entanglement concentration, the highest fiber alignment corresponds to moderate collector rotational speeds. The average fiber alignment maximum value shifts to lower collector rotational speed values with increasing polymer solution concentration, namely, fibers with higher average diameters tend to require significantly lower uptake speed on the rotational collector to achieve a high degree of alignment. In all cases, the highest collector rotational speeds contribute to lower fiber alignment.

A proposed explanation for this phenomenon is that lighter fibers, with smaller average diameters, require a higher uptake speed to be drawn closer to the direction of collector rotation, as well as to overcome bundling and fiber–fiber interactions causing them to entangle near the surface. Conversely, too high a rotational speed of the collector might cause fibers to disperse due to the influence of highly turbulent airflow in the last stages of deposition. This might explain why heavier, larger, straight fibers reach high degrees of alignment even with low collector rotational speeds, but still are prone to being blown to the sides if the collector rotates too quickly.

#### 3.2.4. Static Cylindrical Collector and Static Plate Collector

**Figure 11:**
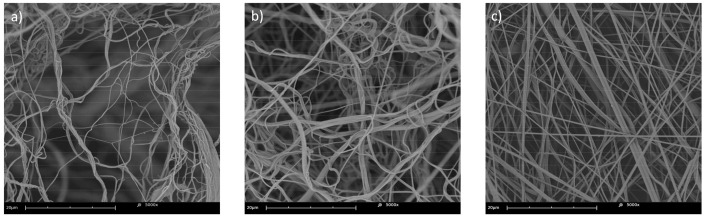
Selected scanning electron microscopy images of the fibrous sample surface, as deposited on a stationary collector, spun from PCL solutions with (**a**) 4%, (**b**) 6%, and (**c**) 8% concentrations.

Qualitative image analysis was performed to compare the sample morphology for fibers gathered on a stationary cylindrical collector and a stationary plate collector. Samples denoted as 0 rpm were made for each PCL 80 kDa solution concentration in the 3–9% range. It was found that fibers collected on a non-moving surface are characterized by a significantly more pronounced “bundling” effect. Fiber bundles are considered characteristic for the solution blow spinning method [73,74]. The effect, however, decreases with an increase in polymer solution concentration, and samples with larger average fiber diameters tend to have straight and randomly aligned fibers. A visual comparison between samples collected on a stationary cylindrical collector for lower and higher PCL solution concentrations—4%, 6%, and 8%—is provided below (Figure 11).

However, it is important to note that samples produced on a static cylindrical collector are relatively small and highly varied in thickness, as only the needle-facing side is covered with fibers. This limits the potential use of a similar collector to produce viable samples and indicates that a larger cylindrical collector, with a different geometry, is needed to obtain a sample with a similar surface area, defeating the purpose of the comparison. In the case of the static metal plate, the fibers tend to gather around plate surface imperfections and to be further deposited around the previously gathered fibers, making the sample visibly uneven in thickness and the fibers oriented towards the closest macroscopic bundle.

## 4. Conclusions

This study showed a method to assess the spinnability window of a polymer–solvent solution and solvent goodness using rheological measurements and proved the method useful for the intended application with qualitative and quantitative image analysis. Critical overlap concentration and critical entanglement concentration were calculated and confirmed with qualitative image analysis. It was found that samples produced from solutions in the dilute, semi-dilute unentangled, and semi-dilute entangled polymer solutions are in the form of polymer film, beaded fibers, and uniform fibers, respectively. The results and conclusions from the rheological approach are consistent with extensive qualitative scanning electron micrograph assessment, lending further validity for potential future use of the proposed approach in similar cases. The influence of the SBS process parameters—polymer solution concentration and collector rotational speed—on fiber diameter, alignment, and morphology was quantified and discussed. A hypothesis was presented to explain the relationship between fiber alignment and polymer solution concentration, as well as collector rotational speed.

The presented work reinforces the position of SBS as a promising method for the production of highly tailorable non-aligned and aligned submicron polymer fibrous scaffolds for varying tissue engineering applications. Fiber diameter measurements confirmed that fibers produced in this study are in the size range of native ECM proteins, confirming the significant potential of the SBS method for the production of tissue engineering scaffolds.

## Figures and Tables

**Table 1:** Collector rotational speed and corresponding linear speed of a point at the collector surface.

Collector Rotational Speed [rpm]	Linear Speed [m/s]
3000	3.93
4000	5.23
5000	6.54
6000	7.85
7000	9.16
8000	10.47
9000	11.78

**Table 2:** Intrinsic viscosity and Mark–Houwink parameter *a* calculation results.

Mn (kDa)	Intrinsic Viscosity [*η*] (cm^3^/g)	*a*
45	153.8 ± 10.4	1.83 ± 0.16
80	441.2 ± 61.8	

**Table 3:** Result summary for average solution blow spun fiber diameters, diameter distribution coefficient of variation, and zero shear viscosity values measured for their respective PCL solution concentrations.

Polymer Solution Concentration [%]	Zero Shear Rate Viscosity of the Solution, $\;\eta_{0}$ [mPa·s]	Average Fiber Diameter [nm]	Coefficient of Variation for Fiber Diameter Distribution
3	93.7 ± 0.2	198 ± 60	30.3%
4	215.5 ± 0.9	238 ± 60	25.21%
5	558.8 ± 4.9	253 ± 60	25.53%
6	582.4 ± 3.7	261 ± 54	20.69%
7	885.1 ± 7.3	319 ± 73	22.88%
8	1604.5 ± 5.3	395 ± 103	25.08%
9	1973.4 ± 6.2	516 ± 150	29.07%

## Data Availability

The data presented in this study are available on request from the corresponding author. The data are not publicly available due to their large volume.
